# Correction: Contact evolution of dry and hydrated fingertips at initial touch

**DOI:** 10.1371/journal.pone.0308241

**Published:** 2024-07-31

**Authors:** The PLOS ONE Staff

The Data Availability statement for this paper is incorrect. The correct statement is: The anonymized data supporting the findings and the scripts used to generate key figures are publicly available in the Edmond repository at https://doi.org/10.17617/3.LF8H2Q.

The publisher apologizes for the error.
