# Supplementary figures and images for: Contact evolution of dry and hydrated fingertips at initial touch
Source: PLoS One. 2022 Jul 13;17(7):e0269722. doi: 10.1371/journal.pone.0269722 (PMC9278764; doi:10.1371/journal.pone.0269722)

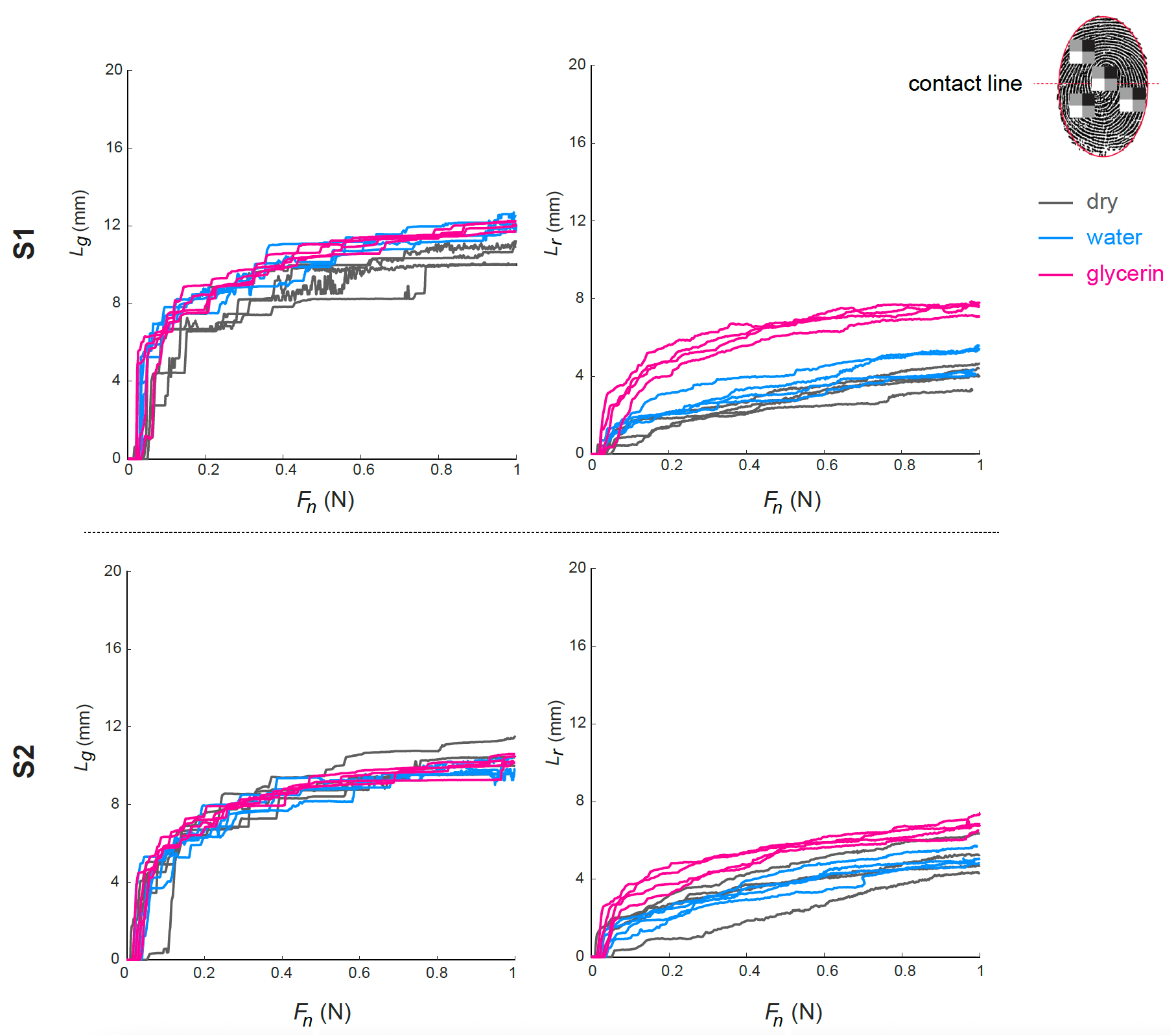

Supplement: S1 Fig — The evolution of gross and real contact lines as a function of applied force for both subjects (S1 and S2 Figs). The results for the three hydration conditions are color-coded. (TIF) [file pone.0269722.s001.tif]

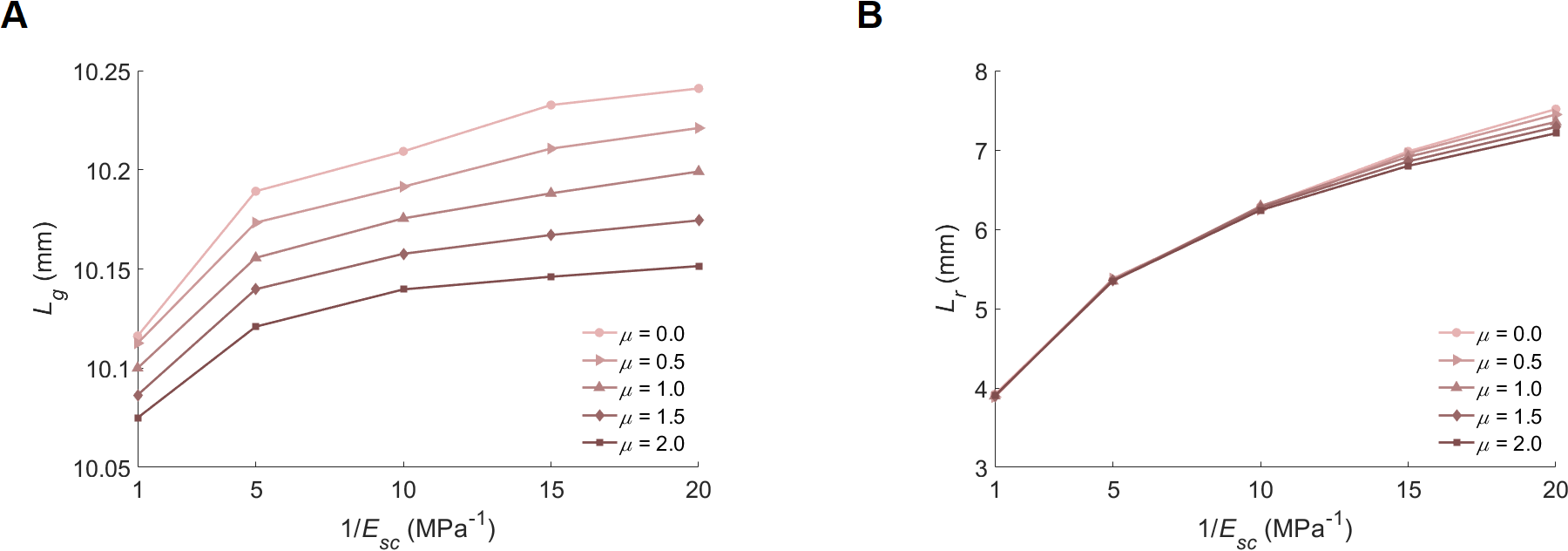

Supplement: S2 Fig — The results obtained for (A) gross and (B) real contact lines are equivalent to the surface plots presented in Fig 4C and 4D, respectively. (TIF) [file pone.0269722.s002.tif]
