# Supplementary material for: Contact evolution of dry and hydrated fingertips at initial touch
Source: PLoS One. 2022 Jul 13;17(7):e0269722. doi: 10.1371/journal.pone.0269722 (PMC9278764; doi:10.1371/journal.pone.0269722)
Supplement: Attachment — Submitted filename: Response to Reviewers.pdf [file pone.0269722.s004.pdf]

Dear Editor and Referees,

We sincerely thank you for your thoughtful and constructive comments on our submitted manuscript. We carefully considered all of your feedback and revised our paper accordingly.

On the pages that follow, *all original reviewer and editor comments are shown in gray italic text.*

- Our responses appear in black upright text that is indented with a bullet at the start. We quote key sections of updated text from the paper; a more detailed log of our edits to the manuscript is provided separately.

After handling each comment from the reviewers, we also carefully edited the manuscript to improve its conciseness, consistency, and flow. These small changes are not described below but are marked in the tracked-changes version of our manuscript.

We believe these changes have made our manuscript significantly stronger, and we again thank the editor and reviewers for their helpful input.

Response to Editor

Both Reviewers highlighted that the study has clear points of merits and can be of interest. Moreover, both Reviewers provided constructive comments for improving the manuscript. Reviewer1, in particular, provided a rather extended set of points that can strengthen both methodology and results (besides the whole presentation flow), thus leading to more robust claims, based on a wider statistical analysis. The Authors are encouraged to leverage the raised comments in order to improve the scientific solidity of the manuscript.

- Thank you for your comments. We carefully edited our manuscript by considering the remarks of both reviewers.

Response to Referee 1

This study quantifies the influence of hydration on fingertip contact evolution, as probed by the gross and real contact area. Those quantities are obtained in vivo from two participants using an optical imaging system monitoring the contact over a transparent surface. It is found that hydration has little effect on the gross contact, but greatly increases the real contact area. The experimental data is then compared to a simulation mimicking the experiment. The authors varied stratum corneum stiffness and contact friction to depict which of those influence the real contact, and found that the stiffness of the SC strongly affects the real contact whereas friction does not.

This is a very interesting study, the measurements made and the comparison to the model are novel and bring new results. The methods are sound and well detailed. The conclusions are well supported by the data.

- Thank you for your concise summary and positive remarks about our work. In the following, we provide our responses to each specific comment that we carefully addressed.

The introduction is hard to follow and misuses several reference references: for instance, some references used for the normal contact are actually experiments on tangential loading and vice versa

(for example ref [9][14][15], at L27). See also comments below. I would recommend the authors to try to improve it.

- Thank you for your comments. We have made several changes in the introduction text and the cited references to improve our scientific arguments and the flow of the text.

Fig2A: please add the corresponding normal force traces. The curves shown in fig 2A are very likely influenced by the speed at which the finger is loaded, it is, therefore, essential to show that the loading dynamics were the same across conditions. Some numbers about the rate (L121-125) are insufficient.

- Thank you for your remark and suggestion. We added the unfiltered normal force traces of both participants to Figure 2A (first column). We also fitted a first-order linear function ($Fn(t) = a(t)+b$) to these traces. The resulting fits have $R^2 > 0.92$ and are shown with the data in Figure 2A (first column); the values of the best-fit slope coefficients appear in Figure 2B. A general linear mixed model showed that the loading force slopes were not significantly affected by the tested conditions. We edited the related paragraphs accordingly, as well as the caption of Figure 2.

The data was obtained from two subjects. Given the variability of the results across subjects, it would be valuable to double or triple the sample.

- Thank you for your suggestion. We agree that taking additional measurements from different subjects would be appropriate for future investigations, and we have added this statement to the discussion. However, we do not believe additional measurements are needed for this manuscript because our main deductions hold despite the variability across the subjects. In addition, the author responsible for conducting the experiments moved to another country and started a new position. Hence, we are unfortunately unable to collect more data in a reasonable time frame. We believe that the manuscript has been significantly improved with the resolution of other issues raised by the reviewers, and we hope that our results will be regarded as sufficient in their current form.

The results related to the model should be presented more coherently with respect to the experimental data. This part lacks clear quantification. Can't you fit the data to the same eq as the experimental data (eq 1), and compare the effect of friction and softening in the model to those observed in vivo (k and m coefficients)?

- Thank you for this good idea. As suggested, we fitted Eq (1) to the simulation results. We added the fits into Fig. 4A-B and presented the corresponding coefficients in the new Table 1. Explanations about the values were also added to the associated paragraph.

Moreover, the 3D plots are hard to interpret.

- Thank you for your remark. We added grids and colorbars to the 3D plots presented in Fig. 4C-D to improve clarity. Additionally, we plotted the results as 2D figures and presented them as Fig. S2 within the supporting information.

Please confirm that those t-tests were corrected for multiple comparisons. Why not use an ANOVA test since there are three factors, or even better, using a mixed-effect model and including both subjects in the same analysis?

- Thank you for these remarks. We followed your suggestion and re-analyzed the results using a general linear mixed model; we conducted a sequential Bonferroni-corrected post hoc test afterward to identify significant pairwise differences. We carefully edited the related paragraph in the results:

“General linear mixed models were created to test the fixed effect of hydration condition on *a,* k_p, k_g, m_r, and m_g. The subject number and measurement order were specified as random effects in the analysis. The loading force slopes did not differ across the three tested conditions. The hydration condition significantly affected real contact area parameters (k_r: $F = 56.45$, $p<0.001$ and m_r: $F = 26.22$, $p<0.001$) but not those for gross contact area (k_g and m_g). A sequential Bonferroni-corrected post hoc test showed that the k_r values for the glycerin condition were significantly higher than the other conditions ($p<0.001$). On the other hand, the m_r for the glycerin condition was significantly lower than the other conditions ($p<0.01$), and m_r for the dry condition was significantly higher than the water one ($p<0.01$).”

The discussion should better discuss the limitation of the approach, in particular, the limitation related to the model, which assumes many simplifying hypotheses.

- Thank you for your comment. Our cross-section model is suitable only for analyzing a fingertip that is horizontally pressed into a flat surface with a contact angle of approximately 0°. In addition, for fast contact scenarios, the agreement between the results from the experiments and simulations is likely to deteriorate since the latter does not include inertial or viscous forces. Excessive pressing forces can also worsen the accuracy of FEA since elastic properties of the tissue can change due to stress-induced effects. We added these explanations about the limitations of the developed finite element model to the sixth paragraph of the discussion.

Finally, I would tone down the comments made with respect to the “friction-induced hinging” phenomenon. First, it results from a particular configuration of the model (gradual contact of the fingerprints) that cannot be reproduced in vivo (the fingerprint are continuously increasing the contact). Second, the effect size of the phenomenon seems very small and is not even quantified.

- Thank you for your comments. In the experimental results, the jumps in the contact area vs. pressing force curves are less apparent compared to the simulation results since the actual ridges grow outward from the fingerprint centroid in a spiral-like manner. However, even in the presence of clear jumps (such as in the experimental contact line results) the said phenomenon may not clearly be observed since we cannot precisely control the friction in the experiments, and this was one of the reasons that we performed simulations. The detected phenomenon is relevant to curved and grooved elastic bodies developing contact. The strength of the induced effects is likely to depend on the elastic properties and the geometry of the object as well as the friction level. Even with a minor influence, this phenomenon can be of interest not only to haptics researchers but also to researchers who study soft contact more generally. Hence, we believe that it is worthy to mention it as an additional observation. Nevertheless, we toned down our related arguments in the last paragraph of the discussion.

Other minor points:

L3-4: not sure those are the right references to support the statement

- Thank you for your remark. We edited the references to support this statement.

L11-13: frictional force? Or friction coefficient?

- Thank you for your question. In this particular sentence, we believe that “force” is the appropriate term because the actual deformations are caused by the applied forces. The friction coefficient provides a means of relating the normal and tangential forces that occur. Thus, we did not change the text on these lines.

L16: ref 8 is about tactile sensors, not about skin.

- Thank you for your remark. With the mentioned reference, we wanted to mention the resistive effect of friction against stretching, which occurs for both synthetic materials and skin. However, since the paragraph is exclusively about human skin, we replaced it with another study by Willemet et al. For similar reasons, the former reference [6] was also replaced by the new references [7] and [8] at the end of the first paragraph.

L21: maybe add Fingerprint ridges allow primates to regulate grip, Yum, et al. PNAS 117 (50), 31665-31673

- Thank you for your comment. Within that sentence, we now cite the suggested study, which was already included in our references.

L24: [15] is also during the onset of slip

- Thank you for your remark. We edited the end of the sentence as "immediately before and during tangential slip [15, 18]" since the onset of slip was primarily investigated but the imaging briefly continued after the slip in both of these studies.

L27: How about Initial contact shapes the perception of friction, Willemet et al. PNAS 118 (49)

- Thank you for pointing out this relevant study. We now cite it as reference [8] in the second paragraph of the introduction.

L71: "The simulation results showed that the elastic moduli influence both gross and real contact area more significantly than friction". It is unclear here why would friction influence real contact.

- Thank you for your comment. In our study, we aimed to rigorously investigate any possible influence caused by the alteration of elastic modulus and friction. Hence, we refrained from making any assumptions based on intuition. In addition, a change in the friction was shown to alter the real contact area in normal touch (Willemet et al. PNAS 118).

L75: "Around these force values, we also detected an interesting physical phenomenon that we name friction-induced hinging, which causes the gross contact line length to be momentarily greater than the one observed for lower friction", This is very unclear at this point, even if it is explained later on.

- Thank you for your remark. We edited that part to improve clarity.

L84: "at a fixed contact angle": please give a value

- Thank you for your remark. The contact angle was approximately 0° in all measurements, and this information has been added to the indicated sentence.

L105: "The resulting fits (R 2 > 0.93) appear with the data in Fig 2A". I am not sure that is true.

- Thank you for pointing out that we had accidentally omitted the fits in the submitted figure file. We added the resulting fits in Fig 2A.

L115: "Independent t-tests showed that the k r values for the glycerin condition were significantly higher than the other conditions for both subjects (p < 0 . 05)". See major comment.

- Thank you for your comment. As explained earlier, following your suggestion, we analyzed the results using a linear mixed model.

L181-187: This part lack of clear quantification. Can't you fit the data to the same eq as the experimental data (eq 1), and compare the effect of friction and softening in the model to those observed in vivo (k and m coefficients)? Moreover, the 3D plots are hard to interpret.

- Thank you for your comments. We addressed these points above as we responded to the major comments.

L208-210: "The absence of the liquid bridges could be explained by the evaporation of water via the microfluidic capillary evaporation mechanism of the fingerprint ridges [7,19] (see condensation in raw images in Fig 2)." This is worrying, how did you compensate for it?

- Thank you for your comment. The misdetection of condensation as finger contact was avoided using the local adaptive threshold methodology. First, the images were smoothed by a Gaussian filter to reduce noise. Then, the local average in a square window with a width of 20 pixels was computed for each pixel in each image. High local average and low local variance indicate the background pixels, which do not contain finger contact. A binarization operation was conducted to identify contact pixels (black) using the local average map as a pixel-wise threshold on the foreground image. We referenced a study (Mehtre and Chatterjee, 1988) that explains this methodology and added the following sentence to the related paragraph for clarity:

  "This local adaptive threshold methodology prevented the misdetection of condensation (see Figure 1A) as finger contact."

L321: "The contact force vector collected in the normal direction was first low-pass filtered by a third-order zero-phase Butterworth filter with a cut-off frequency of 0.5Hz. Then, it was resampled at the frame rate of the camera using interpolation." Cutoff frequency seems extremely low, please justify.

- Thank you for your remark. In our experiments, the participants actively pressed the glass screen with the help of a visual indicator. Hence, the recorded normal force data is slightly affected by finger trembling, causing subtle vibrations in the measurements. By conducting a preliminary analysis using unfiltered force data, we observed that the contact area is not significantly affected by this trembling. Hence, to increase the clarity of the figures, we used the filtered force data for evaluating contact area evolution under different hydration conditions. We edited the related paragraph in the methods section. In this revision process, we double-checked our analysis, and we realized that we actually used a cut-off frequency of 1 Hz, not 0.5 Hz as stated in the original manuscript. The updated text states the correct frequency, and we also added the unfiltered normal force traces for each measurement.

Response to Referee 2

This manuscript presents very nice work on the evolution of contact as a finger pad presses against a flat, rigid surface. Results are presented for both real measurements and simulations, under different conditions of hydration. Real measurements are taken using a sophisticated apparatus and image processing that detects contact by frustrated total internal reflection. Skin hydration is varied by either applying no additional hydration or applying a drop of water or glycerin. A similarly sophisticated finite element simulation is used to measure contacts along a line for a wider range of friction conditions. The results of both approaches are in agreement, and complement each other. Interesting findings are reported, including a new phenomenon that the authors call friction-induced hinging.

I'm impressed by the quality of the work as well as its clear and detailed presentation. The manuscript presents a substantial amount of work, including the development of sophisticated methodologies for both real measurement and simulations. The work is rigorous and well analysed. The prior work is clearly described. The discussion is reasonable and clarifies the implications of the findings and the connections between the real and simulated results. I think this is an excellent paper and I have very few improvements to suggest.

- Thank you for your concise summary and positive remarks about our work.

It would be interesting for the reader to better understand the significance of water and glycerin as hydration mechanism for the fingerpad. I assume, for example, that the hydration of the fingerpad often varies due to the presence or build-up of sweat. Would sweat be considered closer to water or glycerin?

- Thank you for your comment. By following your suggestion, we added the following sentences to the introduction to better emphasize the importance of water and glycerin for the hydration mechanism of the fingerpad.

"These conditions were selected to match dry and moist finger interactions that humans experience daily. For example, humans frequently interact with water (H_2O) when washing their hands, showering, and doing dishes. Sweat, which is naturally excreted from the skin and has been shown to regulate finger grip [6, 18], also substantially contains water. Moreover, glycerin is a non-toxic chemical compound ($C_3H_8O_3$) generally used in cosmetic creams, soaps, and other pharmaceutical skincare products to moisten dry skin. "

Are there other situations of practical interest, such as having oily fingers as noted in the discussion?

- Thank you for your question. We added additional examples to the related sentence in the discussion:

  "This information signals another parameter to consider for understanding human grip and suggests that humans may vary their force control strategies to ensure stable grip when their fingers have been exposed to different compounds (e.g., water, sweat, soap, hand cream, sanitizer, oil). It also gives valuable insights for the design and control of future soft artificial fingers. "

Line 242: "can also observed" is missing a "be".

- Thank you for catching this typo. We added the missing "be".

Figure 2A: Should the labels be S1, S2 instead of SA, SB?

- Thank you for pointing out this error. We corrected the labels to now say "S1" and "S2".
